# Extending calibration-free force measurements to optically-trapped rod-shaped samples

**DOI:** 10.1038/srep42960

**Published:** 2017-02-21

**Authors:** Frederic Català, Ferran Marsà, Mario Montes-Usategui, Arnau Farré, Estela Martín-Badosa

**Affiliations:** 1 Optical Trapping Lab – Grup de Biofotònica, Departament de Física Aplicada, Universitat de Barcelona, Martí i Franquès 1, 08028 Barcelona, Spain; 2 Institut de Nanociència i Nanotecnologia (IN2UB), Martí i Franquès 1, 08028 Barcelona, Spain; 3 Impetux Optics S.L., Trias i Giró 15 1-5, 08034 Barcelona, Spain

## Abstract

Optical trapping has become an optimal choice for biological research at the microscale due to its non-invasive performance and accessibility for quantitative studies, especially on the forces involved in biological processes. However, reliable force measurements depend on the calibration of the optical traps, which is different for each experiment and hence requires high control of the local variables, especially of the trapped object geometry. Many biological samples have an elongated, rod-like shape, such as chromosomes, intracellular organelles (e.g., peroxisomes), membrane tubules, certain microalgae, and a wide variety of bacteria and parasites. This type of samples often requires several optical traps to stabilize and orient them in the correct spatial direction, making it more difficult to determine the total force applied. Here, we manipulate glass microcylinders with holographic optical tweezers and show the accurate measurement of drag forces by calibration-free direct detection of beam momentum. The agreement between our results and slender-body hydrodynamic theoretical calculations indicates potential for this force-sensing method in studying protracted, rod-shaped specimens.

Trap calibration has become routine in force measurement studies involving spherical objects, for which the optical restoring force is well understood^1^. This method can also be applied to non-spherical specimens, with synthetic microbeads bound to the sample of interest used as force probes. This has enabled numerous investigations, such as those into biopolymer stretching^2^, the assembly dynamics of microtubules^3^, cell membrane mechanics^4^ and parasite flagellar forces^5^. However, when it comes to the direct trapping of the sample non-invasively, light momentum transfer gives rise to trapping forces that are difficult to quantify. This, together with complicated hydrodynamic theorizing, makes force calibration for non-spherical samples a complex challenge.

Many microobjects found in nature display cylindrical symmetry, such as rod-shaped *Bacillus* bacteria, *Synedra* and *Nitzschia* diatoms, and eukaryotic nuclear chromosomes. Often, the rod-like shape confers a biological advantage over the spherical form. For example, the larger area to volume ratio of non-spherical mitochondria favors diffusion and makes aerobic respiration more efficient^6^. Likewise, light absorption is maximized in chloroplasts, favoring photosynthesis in plant palisade cells^7^. Direct trapping and measurement of optical forces on rod-shaped specimens is, thus, an important area of interest in several scientific fields.

One solution that is widely applied in bacterial swimming studies consists of monitoring the trapping laser light with a photodiode and inferring information after complex processing of the electric signals obtained. Although not strictly measuring forces, this strategy has successfully shed light on several motility parameters, such as body and flagellar rotation frequencies, velocity variations and direction reversals, cell viability, bacterial swimming patterns and bacterial chemotaxis^8^,^9^,^10^,^11^.

For quantitatively measuring trapping forces on microrods and biological rod-shaped samples, several calibration strategies have been developed, whereby force values are obtained from primary variables such as the sample escape velocity or position^12^. Escape velocity measurement, together with the trap power recording, has enabled the assessment of, for instance, trypanosome swimming forces^5^ and chromosome motility^13^. Meanwhile, the force-position relationship, i.e. the trap stiffness *k*, has been measured for *Escherichia coli* using Stokes’ drag force calibration^14^ and equipartition theorem^15^.

Rod-shaped samples, even if stably trapped in a single Gaussian focus, tend to align their longest dimension with the optical axis due to the gradient force^16^, thus remaining in a position that is not always helpful. To manipulate, rotate and orient them in a specific direction, several coordinating optical traps are necessary. Such multi-trap configurations have been modelled by a stiffness matrix whose calibration permit 3D force and torque measurements^17^,^18^. Experimentally, the trap stiffness matrix of an elongated diatom trapped with two optical tweezers has been obtained from thermal motion analysis for force probing purposes^19^. Likewise, synthetic microrods^20^ and microdevices inducing complex force fields^21^ in a double-trap configuration have been calibrated against Stokes drag. Another technique for stable trapping and orientation of anisotropic samples is the creation of object-adapted optical potentials^22^,^23^, which can be calibrated for force sensing through back-focal-plane interferometry^24^.

In all these cases, precise measurements of the primary variable –escape velocity, trap power or sample position– is of the utmost importance, which, together with the accurate modeling of the sample hydrodynamics^25^,^26^, is very challenging even for regular microrods. Any deviation from the actual sample shape with respect to the geometry conceptualized, as well as uncertainties regarding model approximations or linear assumptions, will produce large inaccuracies in force measurements.

Direct detection of beam momentum enables force measurements without the need for specific trap calibration, as the force is determined directly from the light momentum exchange between the trapping beam and the sample^27^,^28^,^29^,^30^,^31^. Therefore, direct measurement of momentum in non-spherical samples can be performed without complex theoretical calculations. Furthermore, the individual changes in momentum exerted on several optical traps by a sample are added up automatically in the detector, thus directly providing the collective force applied to the trapped object. These advantages come at the cost of complex technical implementation, since all the light creating high-NA optical traps, and therefore contributing to the total momentum exchanged with the sample, must be captured and conveyed to the detector^32^. This is feasible with microbeads and single tweezers, as we showed in refs 28 and 29, but this certainly cannot be taken for granted for rod-like samples or multiple light foci.

In the present study, we measured the total drag force exerted on an arbitrary multiple-bead system trapped by several holographic optical tweezers (HOTs). We then focused on optical manipulation of synthetic microcylinders in a double-trap arrangement and ensured that all the light interacting with the sample was captured. Finally, we compared the drag force measurements with theoretical predictions and discussed the trapping force profiles of dielectric microcylinders.

## Results

### Direct detection of beam momentum

**Figure 1:**
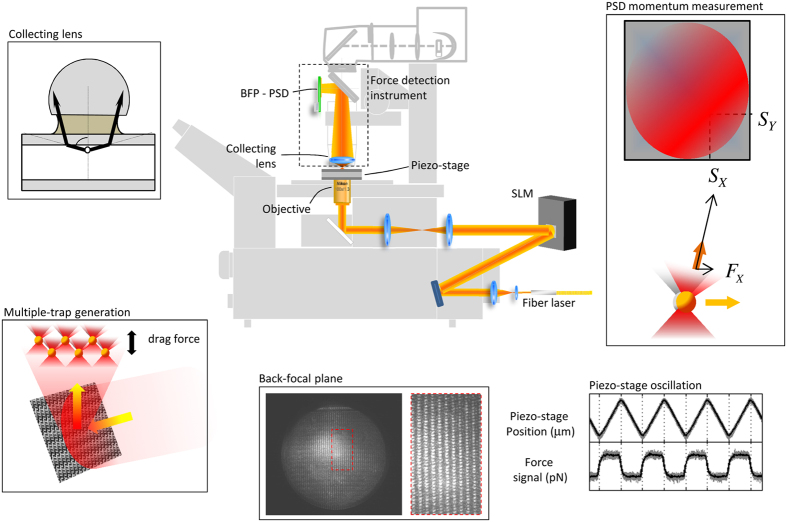
Force measurements based on direct detection of beam momentum changes. The capture of all the light interacting with the trapped specimen by a high-NA lens, provided that it is mostly scattered forward, enables transverse momentum measurements. A PSD at the BFP of the collecting lens yields positional signals, *S*_*X*_ and *S*_*Y*_, proportional to the external lateral force acting on the trapped samples, regardless of the beam structure or object shape and nature, thereby avoiding the need for *in-situ* trap calibration. The spatial light modulator (SLM) creates multiple-trap patterns for the multiple micro-bead experiments. A camera placed on a plane conjugate to the BFP showed the interference of the different light foci. The piezo stage performs a triangular oscillation so as to produce constant force timeframes during which the mean force values are calculated.

The optical force exerted on a trapped sample can be determined from the global change in the linear momentum of the trapping beam, which can be precisely measured at the BFP of a lens entirely capturing the light emerging from the traps. In our set-up, this principle was applied to measure lateral trapping forces, using a position-sensitive detector (PSD) that integrates the overall transverse component of the light beam’s linear momentum. Under specific conditions, discussed in detail in refs 28 and 29, the positional signals of the sensor, *S*_*x*_ and *S*_*y*_, are directly related to the trapping forces, as *F*_*x,y*_ = −*α* · *S*_*x*,*y*_ (see Fig. 1). The volt-to-piconewton equivalence factor *α* is only determined from the geometrical parameters of the set-up – the system focal length *f’* and the sensor radius *R*_*D*_ – and the sensor sensitivity *ψ*. As a result, force measurement based on beam momentum detection is independent of any local parameter present in the experiments, e.g., the laser power, beam structure, objective numerical aperture (NA), and sample geometry and refractive indices. A detailed protocol for the design of a light momentum force sensor can be found in ref. 32.

Correct implementation of this method requires the collection of all the light interacting with the sample. Briefly, this is achieved using a lens of NA higher than the medium refractive index, *NA* > *n*_*medium*_, which enables all the forward scattering 2π square radians of light to enter the lens and reach the PSD, as shown in Fig. 1. We previously showed that the percentage of light backscattered from the sample, which is disregarded by the detection system, was below 3% for common dielectric microspheres in a Gaussian optical trap, producing errors of a similar size in the force readings^28^. However, for more complex objects or sample configurations, it is not clear if backscattered light represents a small fraction of the total light detected or whether it contributes to discrepancies in beam momentum measurements.

In the present study, beam momentum detection was able to measure the collective force generated by multiple-trap systems, which are of interest, for example, in the stable trapping of cylindrical samples. We measured the drag force exerted on multiple microbeads (for which backscattered light was controlled) trapped in an array of holographic optical tweezers (see Methods). The flow oscillation induced by a piezo electric actuator generates drag forces that are compensated by the optical traps, producing beam momentum changes visible at the BFP. It is worth noting that light emerging from each of the optical traps interferes and yields global momentum variation (see Fig. 1), meaning that single bead-trap contributions cannot be extracted. Furthermore, the total force cannot be indirectly inferred from individual bead-trap stiffness calibrations, especially when trapping microspheres of diverse sizes and refractive indices, as each one experiences different drag forces and degrees of optical trap stiffness.

**Figure 2:**
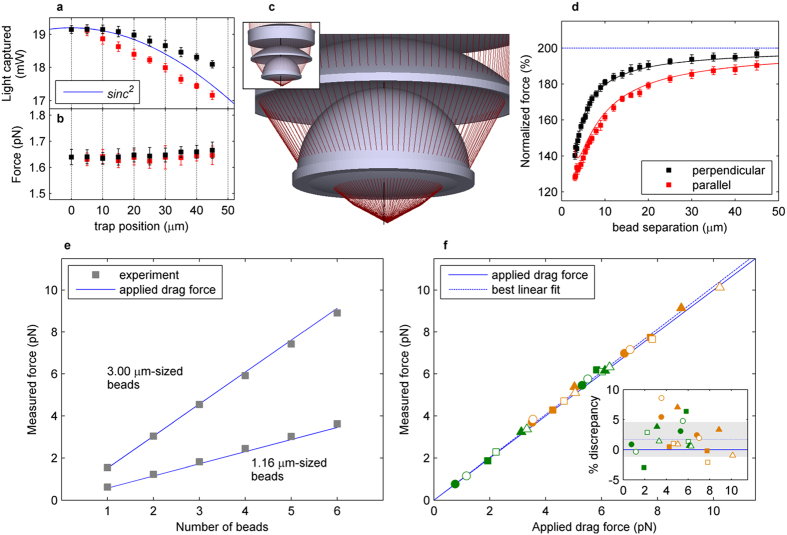
Force measurements on a multiple-bead system. (**a**) Light intensity captured for an empty trap at different positions from the center, which overlaps with the *sinc*^2^ modulation originating from the SLM pixel structure. (**b**) Force measured on a 3.00-μm polystyrene bead in a medium flowing at 60 μm/s, at the same positions as in **a**. Force measured was independent of the bead-trap position with a standard deviation of 0.6%. The black and red symbols show results from the trap steered perpendicularly and parallel to the flow, respectively. (**c**) Ray optics simulation of the set-up capturing all the light from the trap displaced laterally hundreds of microns. (**d**) Forces measured on a two-3.00-μm polystyrene bead system at a 60-μm/s flow against the separation distance. Both traps have a power of 10 mW. The black and red lines show results from the beads separated perpendicularly and parallel to the flow, respectively. Theoretical curves inferred from ref. 35 are superimposed as continuous lines. The blue line illustrates the direct addition of the individual forces. (**e**) Measurements on a system of identical microbeads in a medium flowing at 60 μm/s. The blue lines correspond to the theoretical values. (**f**) Measurements from the twenty-four combinations of beads given in Supplementary Table 1, immersed in a medium flowing at 80 μm/s. The solid blue line is a curve with a slope of one. The dashed blue line is the best linear fit to the data, with a slope of 1.017 and a normalized root mean square error (NRMSE) of 2.9% (shadowed area in the inset).

Total integration of the transverse linear momentum of the trapping beam is derived from the *S*_*X*_ and *S*_*Y*_ channels of the PSD, while the *SUM* signal yields a direct reading of the beam intensity. Using the latter signal, we recorded the power of an empty trap steered holographically along *X* and *Y* (Fig. 2a) to show that there was no loss of light resulting from the position of the optical trap in the trapping plane. That is, we found decreased intensity for off-center traps merely due to diffraction of the pixilated SLM. The same decrease was observed when determining trap stiffness through power spectrum calibration^33^ (data not shown). We additionally applied a 60-μm/s flow to induce a drag force of 1.6 pN on a 3.00-μm polystyrene bead trapped at various sites, confirming that the measured force could be reproduced independently of the trap position (Fig. 2b). Furthermore, light loss in this system was calculated to be less than 1% using a ray optics simulation of the detection set-up (Fig. 2c). Thus, beam momentum detection was robust against a transverse displacement of the trap from the optical axis.

The use of microspheres allows the comparison of our direct force measurements with theoretical values derived from adding all the individual Stokes’ forces together as follows:


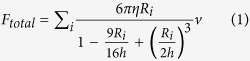


where *R*_*i*_ are the different radii of the microbeads, *v* is the flow velocity, *η* the fluid viscosity whose dependence on temperature is taken into account^34^, and *h* the distance to the upper surface to include Faxén’s effect^1^. In this equation, inter-particle hydrodynamic interactions are not considered and the total drag force on a set of several samples is simply the total sum of single-bead drag forces. To determine to what extent this was valid in our experiments, we trapped two identical 3.00-μm microbeads and recorded the total force as a function of the distance between them (Fig. 2d). At infinity, the total drag force is twice that for a single microbead, decreasing considerably as the microbeads approach each other. Our measurements matched the theoretical curves inferred from ref. 35, allowing for correction due to the presence of a secondary bead. In the six-trap array we subsequently used (Fig. 1), drag force reduction due to interactions was less than 5% for 3.00-μm beads and negligible for smaller microbeads (a detailed explanation of the HOTs array layout is in the Methods section).

We next measured the drag force on a system consisting of identical polystyrene microbeads, either 1.16 or 3.00 μm in diameter, at a flow velocity of 60 μm/s. The force values increased sequentially with the trapping of an additional bead in the array, in accordance with the assumption that the total force is proportional to the number of beads (Fig. 2e). For both bead diameters, results were indeed arranged along a straight line whose slope represents the force per single bead. We performed a similar experiment using the same array of six optical traps, but with microbeads of different sizes and materials. The theoretical drag forces were calculated from the direct addition of individual forces, which were now different for each type of microbead. The measurements, represented against the theoretical values for twenty-four different combinations of microbeads, settled along a straight line with a slope of one (Fig. 2f, see Supplementary Information for details).

These results demonstrated that the light emanating from a set of optical traps can be completely captured and analyzed to determine the total momentum change giving rise to trapping forces. The measurement of momentum was not subject to the specific characteristics of the sample under study – bead sizes and refractive indices – but was defined by a persistent macroscopic calibration, represented by the equivalence factor *α*. It should be noted that a global optical potential describing each of these situations, from which position measurements could hypothetically lead to indirect force calculations, is non-existent if based on the detection of the trapping laser light. The trapping stiffness corresponding to every bead-trap pair should be calibrated separately, for example, by fitting the power spectrum obtained from high-rate video tracking^36^.

Under a certain interpretation, a system of multiple microspheres handled as a whole can also be considered an early irregular object. Light arrives at the detector scattered from different particles at different spatial locations to generate a complicated pattern at the BPF (Fig. 1). Moreover, the holographically modulated laser beam focusing at six different positions at the sample plane stops being purely Gaussian, but can still be collected in its entirety and the overall interchange in momentum measured.

### Force measurements on glass microcylinders

Beam momentum detection can be applied to measure forces on optically trapped cylinders, without the need for previous trap calibration or complete understanding of the trapping dynamics. As already mentioned, once the light collecting system has been set up, accurate measurement depends on the capture of all the light interacting with the specimen. In our drag experiments, two traps were required to hold the cylinders on a plane perpendicular to the optical axis (see Supplementary Movie 1). Therefore, we first studied if nearly all the light creating the two traps would leave the sample and penetrate the collecting lens. Positioning the traps far from the optical axis did not produce significant light loss per se (Fig. 2a and c), leading to the question of whether the particular cylindrical shape contributed to a substantial drop in the captured light due to unfavorable backscattering.

**Figure 3:**
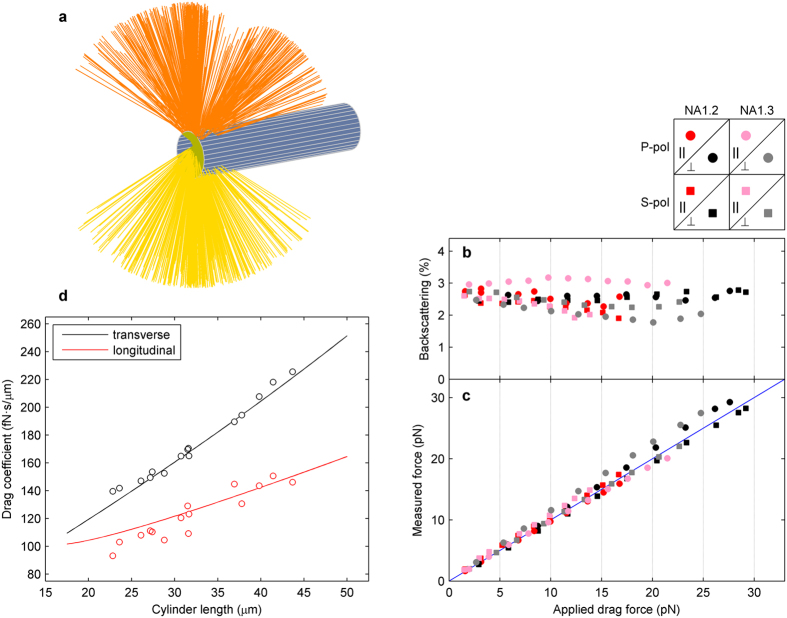
Force measurements on optically-trapped microcylinders. (**a**) Ray optics *OTGO* modeling of the trapping beam acting on the cylinder edge. (**b**) Light scattered backwards for a 33-μm cylinder trapped with two 20-mW optical tweezers, dragged at several flow velocities up to the escape force. The eight data series correspond to *p* and *s* polarizations, water and oil immersion objectives, and transverse and longitudinal drag. (**c**) Forces determined through beam momentum detection over the same data series in **b** matched with theoretical Stokes’ values (solid line), with an NRMSE of ±8%. (**d**) Longitudinal and transverse drag coefficients determined by force measurements on a variety of cylinders, compared with the theoretical values (NRMSE is ±2.6 and ±6.6% for transverse and longitudinal drag experiments, respectively).

Since the microcylinders were far larger than the laser wavelength (20 to 50 μm in length and 5 μm in width), ray optics was used to describe their interaction with the trapping beam (Fig. 3a). We used the *Optical Tweezers in Geometrical Optics* (OTGO) package in Matlab^37^, which computes optical trapping forces and torques from a ray-optics perspective. Interestingly, the user can split the beam resulting from the interaction with the sample into rays travelling forwards and backwards, hence providing an immediate means for determining the amount of light captured by our beam momentum detection instrument (see Methods). For an objective numerical aperture *NA* = 1.2, more than 97.5% of light propagated towards the positive axial direction, slightly decreasing to 96.4% for *NA* = 1.3. These results were consistent with our subsequent measurements.

We performed a Stokes’ drag force experiment for a cylinder trapped in a two-tweezer set-up with a water immersion objective of *NA* = 1.2, and then with an oil immersion objective of *NA* = 1.3. Different flow rates were applied until the cylinder escaped from the traps, both along the transverse and longitudinal directions. Experiments were also carried out for both *p* and *s* polarizations. We determined for each applied flow the amount of light collected in sync with the optical force experienced by the cylinder so as to counteract the drag force (Fig. 3b and c). Backscattering was observed to account for, at most, 3% of the trapping beam intensity, which was measured by removing the cylinder from the traps to avoid obstruction of the laser beam.

Since the light interacting with the cylinders was almost entirely captured, we expected the beam momentum measurements to accurately correspond to the lateral trapping forces. In Fig. 3c, we compare the measured forces with the drag forces applied, which were calculated from slender-body theory^25^:


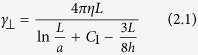



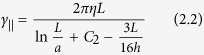


Here, *γ*_*⊥*_ and *γ*_*||*_ are the transverse and longitudinal drag coefficients of the cylinder, such that, at flow velocity *v, F*_*⊥,||*_ =*  γ*_*⊥,||*_*v*. The parameter *η* is the liquid viscosity, *h* the distance to the upper surface, *a* = 2.5 μm is the cylinder radius and *L* its length, which were determined by analyzing bright-field images (see Methods). Meanwhile, parameters *C*_*1*_ and *C*_*2*_ for slender cylinders are defined as: *C*_*1*_ = ln2 − 1/2 and *C*_*2*_ = *C*_*1*_− 1.

During the course of these studies, we observed greater variation in the values compared to similar experiments with microspheres (see refs 28 and 29), which might be attributable to a number of issues. First, most of the cylinders we used had coarse and sharp borders, thus affecting the viscous force. Second, the hydrodynamic model considered assumed high slenderness of the objects under study, which might not be true for the shorter cylinders. Finally, although the cylinders were stably trapped with two optical tweezers, vibration could still occur, thus producing some noise.

We further determined the transverse and longitudinal drag coefficients for a variety of cylinders of different lengths, dividing the force values measured by the applied flow velocity that was now constant for all the samples (Fig. 3d). For both directions, our results and theoretical curves overlapped, with a maximum deviation of ±10%. It should be noted that, again, the same macroscopically determined conversion factor *α* yielded accurate force values from the signals provided by the PSD.

**Figure 4:**
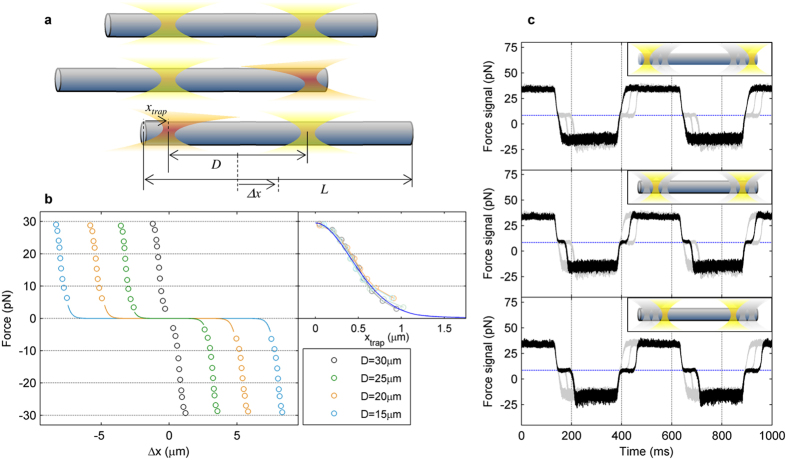
Trapping dynamics of microcylinders. (**a**) Schematic of the trapping dynamics of a cylinder with two holographic optical tweezers. *L* is the cylinder length; *D* is the distance between the two traps. The optical traps exert longitudinal forces only (in orange) when they are close to one of the cylinder edges. (**b**) Experimental force-position curves up to the escape force for a cylinder with *L* = 32.7 μm trapped with two 25-mW optical tweezers and different *D* values. Measured forces are represented against the distance between the cylinder center and the intermediate point between the two traps, Δ*x*. Inset - optical force against the trap position with respect to the cylinder edge, *x*_*trap*_, for the four *D* values. The blue line is the theoretical momentum balance inferred from ray optics (see Methods). (**c**) Force signals for *D* = 30μm (top), *D* = 25 μm (center) and *D* = 20μm (bottom), for *L* = 32.7 μm. Traces for the other two values of *D* are also plotted in light gray for comparison. The dashed blue line indicates the initial momentum of the trapping beam.

Force measurements enabled the assessment of the trapping force profiles (i.e., versus lateral displacement) of the cylinders we manipulated. We determined the longitudinal force profile by combining force detection with position measurements based on video tracking (see Methods). Analogous to the experiments described above, we dragged a given cylinder (*L* = 32.7 μm) by applying a triangular flow oscillation along its longitudinal axis, using four different distances between the two traps, *D*. Consistent with the fact that the optical trap only exerts significant longitudinal forces when interacting with the cylinder ends (see Fig. 4a), we recorded a zero-force plateau for displacements smaller than *Δx*_*0*_ = (*L*–*D*)/2 (Fig. 4b). The force rapidly increases when the first trap reaches the end of the cylinder (the other trap does not interact with the corresponding end as *L* > *D*). The force profile against the trap position with respect to the cylinder edge, *x*_*trap*_, indicates that the optical force profile is independent of *D*.

The time force signals are shown in Fig. 4c. The top and bottom plateaus correspond to the force exerted by one of the traps on their respective cylinder end, whereas the central plateau (when reducing the distance between the traps) corresponds to the time during which the cylinder slides before its rear edge reaches the trap. The central plateau coincides with the initial momentum of the beam, confirming that the cylinder is experiencing no trapping force during this time (see Methods).

Regardless of the *D* value, neither the measured back-and-forth forces nor the initial momentum – corresponding to null force – changed (Fig. 4b and c). Therefore, the measured force only depended on the external drag force applied, which was optically counterbalanced regardless of the trapping beam structure. This reinforces the idea that the direct detection of beam momentum facilitates the optimal choice of the trapping beam or multiple-trap arrangement to manipulate a given sample, providing accurate force measurements without the need for previous *in-situ* trap calibration. In addition, the non-linear response of the force profile did not impede the measurement of optical forces, as they were obtained directly from the detection of changes in the beam momentum.

## Discussion

Direct detection of beam momentum differs from other force-sensing methods in optical micromanipulation, in that force measurements can be obtained directly instead of being inferred from an intricate relationship with the position and orientation of the trapped specimen. Thus, this enables accurate force measurements without the need for specific trap calibration or linearity between the position and orientation of the trapped sample and the optical force.

Accurate measurement requires the capture of all the light interacting with the sample; a condition which is fulfilled as long as light losses are negligible. This has been previously demonstrated for micro-spheres trapped on-axis^28^, as well as in the present study for multiple holographic traps spread over the sample field of view and optical manipulation of cylindrical objects. In the multiple-bead experiment, we demonstrated that the high-NA collecting lens captured all the light from off-axis traps, and that the force was independent of the trap position. Backscattered light for cylinders was assessed to be 3% of the incoming light, despite their particular symmetry.

The macroscopic calibration of the set-up, represented by the volt-to-picoNewton parameter *α*, is not affected by local variables necessary to calibrate traps *in-situ*, such as temperature, viscosity and trapped object geometry. Likewise, it does not depend on other key parameters determining optical trapping dynamics: laser power, trapping beam NA and structure, and medium and sample refractive indices, among others. Furthermore, force measurements can be undertaken on non-spherical samples^17^,^18^ and non-viscous media^38^, for which calibration is considerably complex.

In this paper, we have shown that the measurement of the total drag force exerted on a multiple-bead system can be addressed by analyzing the total momentum exchanged between the multiple-spot trapping beam and the several trapped beads. An indirect calibration-based method is not applicable here, as individual bead-trap calibration is inaccessible at the BFP. The system collects all the light creating the different optical traps and measures the change in the transverse component of the beam momentum, which is equivalent to the entire lateral drag force applied. Multiple-spot optical tweezers have been widely used for stable trapping of extended objects and calibrated for force measurements^17^,^18^,^19^,^20^,^21^,^39^. However, the independence of the *in-situ* experimental conditions permits the creation of arbitrary trap patterns adapted to a particular situation without the need of a new calibration.

The same principle can be applied for the determination of axial momentum changes from beam power concentration measurements, which can be carried out either with photo-detectors with specific radially-dependent transmission profiles^40^ or a high-speed camera tracking the trapping beam intensity distribution at the BFP^31^.

We have demonstrated the accurate measurement of hydrodynamic forces on glass microcylinders trapped with pairs of optical tweezers and shown that most light was eventually forward-scattered, which permitted beam momentum measurements. For micro-rods in the same order of magnitude of biological specimens, i.e. in the micron range, we measured 2.1 ± 0.6% backscattering (data not shown). For microorganisms trapped in watery solutions, the relative refractive index is *n*_*rel*_ = 1.04 (e.g. *E. coli* refractive index *n*_*EC*_ = 1.388^41^), hence, there will be negligible backscattering and the light-momentum detection principle can be applied. Therefore, the results here are applicable to precise measurements on rod-shaped organisms in microbiological studies, which constitutes a primary concern in microbiology as they are widely found in nature. For example, in experiments analyzing hydrodynamic properties of biological swimmers, a given microorganism can be trapped with pairs of optical tweezers that can be used for controlled alignment and orientation.

In addition to rod-shaped samples, the method described here can be undertaken in non-viscous media, such as the interior of a cell. Biological cargoes driven by molecular motors can be directly trapped without the aid of spherical probes, and the pulling forces measured under strict physiological conditions. Importantly, many of these cargoes have an elongated, rod-like shape (e.g., chromosomes, mitochondria, peroxisome peroxules, etc.). Even vesicles that are frequently targeted in cellular experiments for their appropriate characteristics, such as lipid droplets, can change from their rigid homogeneous sphere when they increase in size, posing difficulties for the calibration even in controlled conditions *ex vivo*^42^.

The necessity for capturing a significant fraction of the scattered light is not more limiting in biological studies than in the experiments we carried out here. For example, inside a cell, backscattering is reduced as the relative refractive index of intracellular organelles (i.e., with respect to that of the cytoplasm) barely reaches *n*_*rel*_ = 1.1 (e.g., lipid droplet refractive index *n*_*LD*_ = 1.48 − 1.53^43^, cytoplasm refractive index *n*_*c*_ = 1.36 − 1.375^44^), whereas for glass microcylinders in water, *n*_*rel*_ = 1.17. Angular light-scattering studies show that even in organelles with complex internal structure, such as mitochondria, scattering predominantly occurs in the forward direction^45^.

Direct detection of beam momentum can also be used for synthetic objects with interesting trapping properties. As an example, elaborate microprobes exhibiting specifically engineered force fields can be manipulated and quantitatively analyzed, with possible applications for photonic force microscopy. As previously mentioned, the tendency for elongated objects to align parallel to the optical axis, as observed both in biological and synthetic samples, can be easily resolved by using pairs of optical traps without impeding the measurement of global optical forces. Moreover, the arbitrariness of the HOTs array used in the multiple-bead experiment strongly suggests that direct detection of beam momentum is suitable for quantitative experiments with complex, non-Gaussian trapping beams creating adapted optical potentials.

## Methods

### Optical tweezers set-up

The laser beam (*λ* = 1064 nm TEM00, IPG YLM-5-1064-LP) was expanded through a telescope to fit the active area of a reflective SLM (Hamamatsu X10468-03: 800 × 600 pixels) and subsequently readjusted to the objective entrance pupil through another telescope. The beam enters an inverted microscope (Nikon Eclipse TE2000-E) through a rear port and a dichroic mirror reflects it up towards the microscope objective (either water immersion Nikon Plan Apo, 60x, *NA* = 1.2 or oil immersion Nikon CFI Plan Fluor, 100x, *NA* = 1.3), creating the optical traps at its focal plane. Microchambers were placed onto a piezo electric stage (Piezosystem Jena, TRITOR 102 SG). Lateral optical trapping forces, as well as optical trap intensity, were measured by a direct force-detection instrument (Impetux Optics, LUNAM T-40i). This instrument enables the simultaneous collection of the laser light emerging from the optical traps as well as bright-field illumination, hence allowing sample imaging, which was performed at a different rear port with a CCD camera (QImaging, QICAM).

### Sample preparation and manipulation

Microchambers, approximately 90 μm high, were made by gluing together a microscope slide (1 mm thick) and a coverslip (150 μm thick) with double-sided tape in which a 1 × 1 cm cavity was created. In all the experiments, we diluted the samples in water to a considerably low density so as to avoid unexpected trapping. Collective force measurements in multiple-particle experiments were carried out on the following set of synthetic microspheres: 0.61-, 1.16-, and 3.00-μm polystyrene microbeads; 2.19-μm melamine resin microbeads and 2.32-μm silica microbeads (see Supplementary Table 1). For the experiments on cylinders, we used glass microrods (Nippon Electric Glass, PF-50) with a diameter of 5 μm and lengths between 20 and 50 μm. Due to the considerable weight of such samples (a 20-μm cylinder weighs 10 pN), the power at both the two optical traps holding the cylinder needed to be above a threshold of about 10 mW. All measurements were performed at *h* = 20 μm from the upper microscope slide.

### Piezo stage oscillation

The piezo stage was controlled by a LabView software through a NI-DAQ *close-loop* interface. For the drag force to be constant, the piezo stage produced a triangular oscillation (see Fig. 1). Once the flow velocities to be applied were calculated, the frequency of the oscillation was chosen to be as low as possible, bearing in mind the highest amplitude available was ±40 μm. This way, the constant force timeframe was as long as possible. However, actual velocities differed from the desired ones by an amount dependent on the oscillation parameters, amplitude and frequency. This was up to 5%, especially for higher oscillation frequencies. In any case, for the theoretical Stokes’ calculations, we took the actual velocity from the direct reading of the piezo stage monitor output.

### Drag force measurements

Force measurements are affected by Gaussian noise that is averaged out by calculating the mean force value throughout the constant force plateau. The initial momentum of the beam, i.e., the reading of the PSD when no external force was applied, was subtracted by calculating half the difference between the back and forth half-period plateaus of the force square signal. This measurement was repeated twenty times in all the experiments to obtain an accurate average force value and an error bar from its standard deviation. Error bars ranged from 20 fN to 50 fN (from 2% to 5% uncertainty for 1 pN force measurement), which correspond to the measurement repeatability and, thereby, to the force limit detection. This uncertainty was affected by several experimental features, such as laser power and stability, sample steadiness (especially in the cylinder trapping case), among others.

### Multiple-bead trapping

The array of six holographic optical tweezers, generated by Gerchberg-Saxton algorithm, was spread over the available field of view (see Supplementary Figure 1). To minimize hydrodynamic interactions so that the direct addition of all the individual forces would be correct, two aspects were taken into consideration. First, separation perpendicular to the flow oscillation was set to 22.5 μm, at which hydrodynamic interactions yielded less than a 5% decrease in drag force for the larger beads used (3.00 μm in diameter). Second, since longitudinal interaction is considerably higher, separation parallel to the flow was set to 50 μm and shifted 11.25 μm perpendicularly, resulting in a similar force reduction (see Fig. 2d). For all the other beads used, hydrodynamic interaction was smaller or unnoticeable.

### Cylinder tracking

An in-house cross-correlation algorithm in Matlab was implemented to track the trapped cylinder position with one-pixel accuracy, corresponding to 0.08 μm at the sample plane. Cylinder lengths were measured from their bright field images, within an accuracy of 0.4 μm, corresponding to ± 5 pixel variability when determining the cylinder edge.

In Fig. 4b, *Δ*x was calculated as half the difference between the two extreme cylinder positions when being dragged back and forth, in a way analogous to force measurements with respect to the initial beam momentum. These two positions were determined by averaging the corresponding half oscillation period. We then duplicated and used the force measurements to plot the restoring optical force for both positive and negative *Δx* values. In the inset, the position of the left trap with respect to the left cylinder edge was calculated as *x*_*trap*_ = *(L* *−* *D)/*2 − *Δx*. As shown, the four curves – corresponding to different *D* values – overlapped after this transformation.

### Ray optics force simulation

Optical trapping of dielectric cylinders was simulated by a ray-optics approach, using the Matlab toolbox *Optical Tweezers in Geometrical Optics* (OTGO)^37^. Briefly, the trapping beam was modeled as a set of focusing rays, which interacted with the sample in terms of geometrical optics – Fresnel’s and Snell’s laws. Optical forces were calculated by carefully extracting the beam momentum balance of a number of scattering iterations.

For comparison with Fig. 4b, in which longitudinal optical force arises from the trap interacting with the cylinder end, we simulated a single optical trap. The beam specifications were set as *NA* = 1.2 and *f’* = 3.33 mm for the water immersion objective, and *P* = 25 mW was taken as the trap power at the sample (the trap intensity measurement through the PSD sum channel was 50 mW as there were two traps). The beam waist was used as a free parameter for the best fit, which was obtained for *ω*_*0*_ = 1.58 mm.

Light loss was estimated by splitting the rays exiting the trapped sample into those with a negative and positive *z*-component. The latter were captured by the collecting lens since its NA was higher than the medium refractive index (see Results).

## Additional Information

**How to cite this article:** Català, F. *et al*. Extending calibration-free force measurements to optically-trapped rod-shaped samples. *Sci. Rep.* **7**, 42960; doi: 10.1038/srep42960 (2017).

**Publisher's note:** Springer Nature remains neutral with regard to jurisdictional claims in published maps and institutional affiliations.

## Supplementary Material

Supplementary Information

Supplementary Video
