# Supplementary material for: Extending calibration-free force measurements to optically-trapped rod-shaped samples
Source: Sci Rep. 2017 Feb 21;7:42960. doi: 10.1038/srep42960 (PMC5318951; doi:10.1038/srep42960)
Supplement: Supplementary Information [file srep42960-s1.pdf]

Supplementary information

Extending calibration-free force measurements to optically-trapped rod-shaped samples

Frederic Català[1,2], Ferran Marsà[3], Mario Montes-Usategui[1,2],
Arnau Farré[2,3], Estela Martín-Badosa[1,2*]

[1] *Optical Trapping Lab – Grup de Biofotònica, Departament de Física Aplicada, Universitat de Barcelona, Martí i Franquès 1, 08028 Barcelona, Spain (http://biopt.ub.edu)*

[2] *Institut de Nanociència i Nanotecnologia (IN2UB), Martí i Franquès 1, 08028 Barcelona, Spain*

[3] *Impetux Optics S.L., Trias i Giró 15 1-5, 08034 Barcelona, Spain*

[*]*Corresponding author*: estela.martinb@ub.edu

Multiple-bead trapping

As mentioned in the *Results* section, our spatial light modulator uses the Gerchberg-Saxton algorithm to focus the trapping beam at six different locations, hence creating an array of six optical traps. We prepared samples containing five types of spherical microbeads (see Supplementary Table 1) and applied a constant flow rate of 80 µm/s, using a piezo stage to create triangular oscillations.

For twenty-four combinations, we evaluated the collective force acting on the system. The theoretical values were calculated by adding all the individual forces, since the traps were sufficiently separated (see text). Individual theoretical forces were calculated using Stokes' law, taking into account the bead-to-surface interaction (Faxén's law) and water viscosity dependence on temperature.

Combination ID		0.61 µm (PS)	1.16 µm (PS)	2.19 µm (MR)	2.32 µm (Silica)	3.00 µm (PS)	$\sum_i \boldsymbol{F}_i$	Measured force
**1**	● (green)		1				0.76	0.77
**2**	○ (green)	1	1				1.16	1.16
**3**	■ (green)	1	2				1.93	1.87
**4**	□ (green)		1	1			2.23	2.29
**5**	▲ (green)			1	1		3.13	3.25
**6**	△ (green)	1		2			3.33	3.37
**7**	● (orange)	1	2	1			3.51	3.70
**8**	○ (orange)	1		1	1		3.54	3.84
**9**	■ (orange)		1	1		1	4.26	4.28
**10**	□ (orange)	1	1	1		1	4.65	4.70
**11**	▲ (orange)	1	2	2			5.03	5.38
**12**	△ (orange)	2	1	1		1	5.05	5.10
**13**	● (green)		2		1	1	5.30	5.46
**14**	○ (green)		3	1	1		5.50	5.77
**15**	■ (green)	1	3	2			5.82	6.19
**16**	□ (green)		1	1	1	1	6.02	6.10
**17**	▲ (green)	1	1	2		1	6.12	6.16
**18**	△ (green)		1	1			6.28	6.32
**19**	● (orange)		2	1	1	1	6.81	6.98
**20**	○ (orange)		3	2	1		7.02	7.16
**21**	■ (orange)		1	2		2	7.75	7.73
**22**	□ (orange)	1	2		1	2	7.81	7.65
**23**	▲ (orange)			2	1	2	8.85	9.14
**24**	△ (orange)		1	1	1	3	10.23	10.13

Supplementary Table 1 | Multiple bead combinations. For each combination, the number of beads of each kind trapped in the optical tweezers array is indicated (PS: Polystyrene, MR: Melamine Resin, Si: Silica), as well as the theoretical force and the collective force measured. We have not indicated the position of the beads in the trap array as it did not affect the results.

All measurements agreed with the theoretical values expected, with a maximum peak-to-peak variability of 9%. We show an example of a bead-trap arrangement (combination 17) in Supplementary Figure 1.

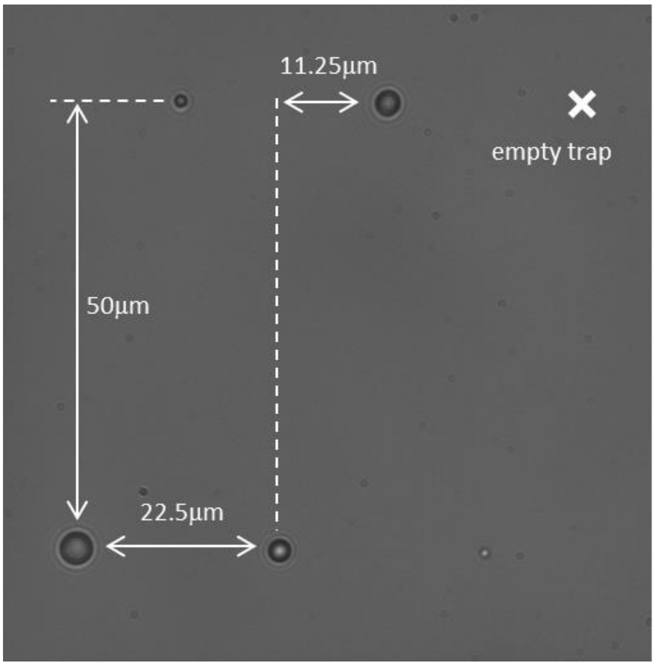

Supplementary Figure 1 | Bright field microscope image of combination 17 (see Supplementary Table 1), indicating the bead separation to minimize hydrodynamic interactions.

Cylinder trapping

In Supplementary Movie 1, we show the trapping of a glass microcylinder with two optical tweezers generated holographically, which allowed stable trapping and the orientation of the sample either longitudinally or transversely to the flow induced by the piezo electric stage.

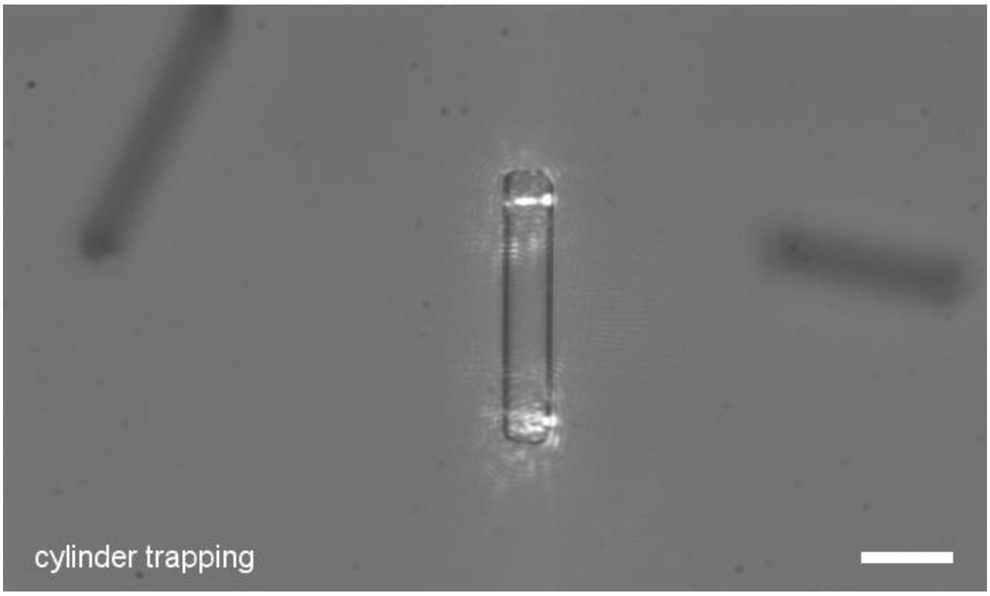

Supplementary Movie 1 | Cylinder manipulation with a pair of optical tweezers. Two cylinders out of focus are laying at the bottom surface, while the central cylinder is stably trapped at the objective image plane, and transverse and longitudinal drag forces are measured. Scale bar: 10 µm. Video played back at 3x speed.
